# “I’M GOING TO LIVE HOW LONG?” EXPLORING GAPS AND INFLUENTIAL FACTORS IN PERCEPTIONS OF LIFE EXPECTANCY

**DOI:** 10.1093/geroni/igae098.3262

**Published:** 2024-12-31

**Authors:** Lauren Cerino, Adam Felts, Lisa D’Ambrosio, Taylor Patskanick, Joseph Coughlin

**Affiliations:** Massachusetts Institute of Technology, Cambridge, Massachusetts, United States; Massachusetts Institute of Technology, Cambridge, Massachusetts, United States; MIT AgeLab, Cambridge, Massachusetts, United States; Massachusetts Institute of Technology, Cambridge, Massachusetts, United States; MIT AgeLab, Cambridge, Massachusetts, United States

## Abstract

As the population ages and life expectancy increases, it becomes increasingly important to understand the public’s perceptions and expectations of their own aging, longevity, and life expectancy. Research shows that most people tend to underestimate their own life expectancy (Bago d’Uva, O’Donnell, and van Doorslaer, 2017; Society of Actuaries, 2020). Having an accurate sense for how long one may live plays an important role in informing decision-making and planning around finances, health, and general aspirations. The MIT AgeLab conducted focus groups (N=69, ages 27-76) and a survey (N=1184, ages 20-79) examining participants’ perceived life expectancy and the factors influencing their expectations. The survey sample’s overall perceived life expectancy was 79.84 years, with differences emerging among demographics; for example, older participants reported significantly higher life expectancy compared to younger participants. Health status, lifestyle, and familial health history emerged as top factors influencing participants’ expectations. Further analysis will explore differences in perceived life expectancy among participants with varying life experiences (e.g., caregiving status, employment, family history), self-reported health, hopes and goals (e.g., desired life expectancy), and personality traits (e.g., optimism), as well as demographic characteristics (e.g., age, gender, race/ethnicity, residential environment). We will suggest both psychological (via construal theory) and sociological (via analyzing sociocultural knowledge about old age as a life stage) explanations for these findings. This session will emphasize practical implications for researchers and practitioners to inform how they might guide individuals toward more accurate perceptions of their life expectancy, enabling people to engage in improved decision-making and planning.

